# Supplementary material for: Solubility Determination and Comprehensive Analysis of the New Heat-Resistant Energetic Material TNBP
Source: Molecules. 2023 Mar 7;28(6):2424. doi: 10.3390/molecules28062424 (PMC10054621; doi:10.3390/molecules28062424)
Supplement: Supplementary file 1 [file molecules-28-02424-s001.zip › molecules-2220721-supplementary.pdf]

Supporting Information

Solubility Determination and Comprehensive Analysis of the New Heat-resistant Explosive TNBP

Luoluo Wang [1,2], Minchang Wang [1,2,*], Ying Kang [1,2], Yong Zhu [1,2], Hai Chang [1,2] and Ning Liu [1,2,*]

1 Xi'an Modern Chemistry Research Institute, Xi'an 710065, China;
2 State Key Laboratory of Fluorine & Nitrogen Chemicals, Xi'an 710065, China;

* wmc204@163.com (M. W.); flackliu@sina.com (N. L.).

Table S1. Calculations of fusion enthalpy of TNBP by group contribution method.

Group	Number	Type	Contribution (kJ·mol^{-1})
$-NO_2$	6	Y	5.89
>CH-	4	ar	1.59
>C<	12	ar	-0.86
>N-	6	ar	2.39
-O-	2	ar	2.39
Total			50.50

Table S2. KAT-LSER model parameters for six pure solvents.

Solvent	polarity[1]	α	β	π*	δ_H^2/1000 (J·cm^{-3})
DMSO	44.4	0.00	0.76	1.00	0.560
DMF	40.4	0.00	0.77	0.88	0.527
NMP	36	0.00	0.77	0.92	0.507
acetone	35.5	0.08	0.43	0.71	0.399

acetonitrile	46	0.19	0.40	0.75	0.581
ethyl acetate	23	0.00	0.45	0.55	0.386

Table S3. Correlation of TNBP model parameters in different solvents using the van't Hoff equation.

Solvent		A	B	100ARD	10^3RMSD	R^2
DMSO		-0.89	-609.07	1.257	0.907	0.9844
DMF		-2.33	-139.44	0.531	0.405	0.9456
NMP		-2.13	-58.13	0.105	0.119	0.9886
acetone		-2.08	-862.43	1.725	0.158	0.9484
acetonitrile		-0.76	-1830.09	5.854	0.097	0.9569
ethyl acetate		-4.22	-445.62	2.074	0.087	0.9038
DMSO + water	ω_{DMSO}=0.80	11.25	-6849.00	20.180	0.003	0.9990
	ω_{DMSO}=0.85	5.37	-4168.31	33.997	0.053	0.9896
	ω_{DMSO}=0.90	7.07	-3918.69	13.297	0.381	0.9952
	ω_{DMSO}=0.95	5.12	-3045.75	10.032	1.313	0.9742
DMSO + acetone	ω_{DMSO}=0.30	-1.69	-633.56	1.434	0.400	0.9382
	ω_{DMSO}=0.50	-1.24	-629.81	1.465	0.639	0.9371
	ω_{DMSO}=0.70	-0.81	-679.52	1.309	0.756	0.9556
	ω_{DMSO}=0.90	-0.85	-636.75	1.466	0.940	0.9359
DMSO + acetonitrile	ω_{DMSO}=0.30	-0.19	-1484.88	3.227	0.273	0.9802
	ω_{DMSO}=0.50	-0.24	-1163.22	2.406	0.535	0.9819
	ω_{DMSO}=0.70	-0.98	-768.88	1.645	0.596	0.9809
	ω_{DMSO}=0.90	-1.82	-355.62	1.221	0.719	0.9508

DMSO + ethyl acetate	ω_{DMSO}=0.30	-2.24	-333.34	0.914	0.384	0.9677
	ω_{DMSO}=0.50	-1.73	-417.69	1.028	0.543	0.9744
	ω_{DMSO}=0.70	-1.38	-484.63	1.583	1.005	0.9516
	ω_{DMSO}=0.90	-1.11	-531.76	1.572	1.056	0.9646

Table S4. Correlation of TNBP model parameters in different solvents using the modified Apelblat equation.

Solvent		A	B	C	100 ARD	10^3 RMSD	R^2
DMSO		-59.98	2206.15	8.72	0.190	0.135	0.9997
DMF		-28.20	1088.43	3.82	0.072	0.055	0.9990
NMP		-6.92	169.11	0.71	0.019	0.025	0.9995
acetone		-92.08	3259.77	13.37	1.238	0.114	0.9737
acetonitrile		-95.80	2673.45	14.03	4.416	0.071	0.9763
ethyl acetate		-134.35	5666.91	19.24	0.482	0.018	0.9960
DMSO + water	ω_{DMSO}=0.80	-134.21	403.50	21.29	32.180	0.004	0.9981
	ω_{DMSO}=0.85	23.93	-5073.81	-2.73	32.952	0.065	0.9903
	ω_{DMSO}=0.90	-58.65	-712.25	9.65	15.921	0.046	0.9929
	ω_{DMSO}=0.95	-109.42	2527.52	16.84	7.289	0.966	0.9861
DMSO + acetone	ω_{DMSO}=0.30	-87.84	3310.07	12.80	0.947	0.262	0.9730
	ω_{DMSO}=0.50	-77.11	2843.36	11.27	1.047	0.448	0.9690
	ω_{DMSO}=0.70	-79.54	2925.50	11.70	0.877	0.497	0.9808
	ω_{DMSO}=0.90	-86.92	3303.49	12.79	0.982	0.630	0.9714
DMSO	ω_{DMSO}=0.30	-188.82	7430.27	27.86	0.966	0.074	0.9985
	ω_{DMSO}=0.50	-145.34	5681.29	21.44	0.252	0.059	0.9998

+ ace-tonitrile	ω_{DMSO}=0.70	-98.55	3823.22	14.42	0.266	0.103	0.9994
	ω_{DMSO}=0.90	-75.77	3116.69	10.94	0.123	0.078	0.9994
DMSO + ethyl acetate	ω_{DMSO}=0.30	-58.28	2297.70	8.29	0.055	0.022	0.9999
	ω_{DMSO}=0.50	-63.38	2478.38	9.12	0.141	0.080	0.9994
	ω_{DMSO}=0.70	-101.04	4198.41	14.74	0.159	0.111	0.9994
	ω_{DMSO}=0.90	-93.49	3810.18	13.66	0.231	0.176	0.9990

Table S5 Correlation of TNBP model parameters in different solvents using the λh equation.

Solvent		λ	h	100ARD	10^3RMSD	R^2
DMSO		0.004	9262.07	0.808	0.572	0.9938
DMF		-0.061	24784.30	0.226	0.175	0.9899
NMP		-0.107	21159.84	0.090	0.101	0.9920
acetone		0.011	46419.68	1.630	0.150	0.9552
acetonitrile		0.023	73994.06	5.570	0.092	0.9599
ethyl acetate		-0.001	222669.58	1.774	0.072	0.9348
DMSO + water	ω_{DMSO}=0.80	2.601	2632.78	20.204	0.003	0.9990
	ω_{DMSO}=0.85	0.407	10230.21	34.177	0.068	0.9896
	ω_{DMSO}=0.90	3.407	1158.51	13.098	0.375	0.9953
	ω_{DMSO}=0.95	1.787	1714.41	10.081	1.327	0.9737
DMSO + acetone	ω_{DMSO}=0.30	0.008	21044.57	1.318	0.367	0.9485
	ω_{DMSO}=0.50	0.012	12935.34	1.353	0.586	0.9471
	ω_{DMSO}=0.70	0.030	8776.26	1.199	0.688	0.9630
	ω_{DMSO}=0.90	0.022	8510.57	1.350	0.867	0.9456

DMSO + acetonitrile	ω_{DMSO}=0.30	0.060	21942.52	3.001	0.251	0.9832
	ω_{DMSO}=0.50	0.073	12646.91	2.113	0.467	0.9862
	ω_{DMSO}=0.70	0.025	13343.76	1.292	0.466	0.9883
	ω_{DMSO}=0.90	-0.033	16586.52	0.871	0.513	0.9750
DMSO + ethyl acetate	ω_{DMSO}=0.30	-0.025	25277.92	0.557	0.234	0.9877
	ω_{DMSO}=0.50	-0.023	16378.76	0.668	0.352	0.9893
	ω_{DMSO}=0.70	-0.017	12401.96	1.220	0.784	0.9706
	ω_{DMSO}=0.90	-0.009	10001.11	1.215	0.812	0.9791

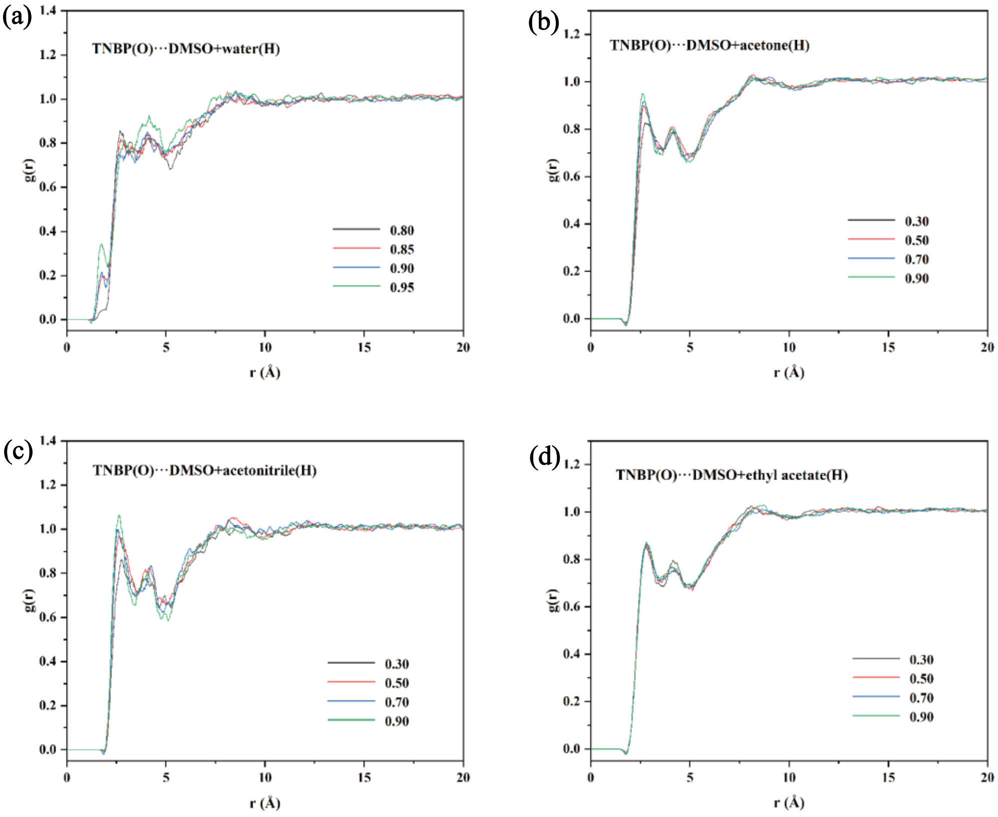

Figure 1S. RDF plots between TNBP and selected mixed solvents at 313.15K.

Table S6 Thermodynamic parameters of TNBP in pure and mixed solvents

Solvent		$\Delta_{dis}H$ (KJ·mol^{-1})	$\Delta_{dis}S$ (J·K^{-1}mol^{-1})	$\Delta_{dis}G$ (KJ·mol^{-1})
DMSO		5.06	-7.40	7.45
DMF		1.16	-19.37	7.40
NMP		0.48	-17.71	6.18
acetone		7.17	-17.29	12.49
acetonitrile		15.22	-6.32	17.22
ethyl acetate		3.70	-35.09	14.83
DMSO + water	ω_{DMSO}=0.80	56.94	93.53	26.83
	ω_{DMSO}=0.85	34.66	44.65	20.28
	ω_{DMSO}=0.90	32.58	58.78	13.66
	ω_{DMSO}=0.95	25.32	42.57	11.62
DMSO + acetone	ω_{DMSO}=0.30	5.27	-14.05	9.59
	ω_{DMSO}=0.50	5.24	-10.31	8.41
	ω_{DMSO}=0.70	5.65	-6.73	7.72
	ω_{DMSO}=0.90	5.29	-7.07	7.47
DMSO + acetonitrile	ω_{DMSO}=0.30	12.35	-1.58	12.85
	ω_{DMSO}=0.50	9.67	-2.00	10.30
	ω_{DMSO}=0.70	6.39	-8.15	8.98
	ω_{DMSO}=0.90	2.96	-15.13	7.76
DMSO + ethyl acetate	ω_{DMSO}=0.30	2.77	-18.62	8.68
	ω_{DMSO}=0.50	3.47	-14.38	8.04
	ω_{DMSO}=0.70	4.03	-11.47	7.67
	ω_{DMSO}=0.90	4.42	-9.23	7.35

```
my $results = Modules->Forcite->Analysis->VelocityAutocorrelationFunction($doc,
Settings(
    VACFSetA => 'TNBP'));
my $outVACFChart = $results->VACFChart;
my $outVACFChartAsStudyTable = $results->VACFChartAsStudyTable;
```
